# Review of Risk Factors for Opioid Misuse and Addiction Following Traumatic Injury

**DOI:** 10.3390/healthcare14050564

**Published:** 2026-02-24

**Authors:** Nicholas J. Lawler, Bipasha Sobhani, Ejura Yetunde Salihu, Hannah Muller, Jordan Edwards, Megan Ringo, Randall Brown

**Affiliations:** 1 Department of Microbiology, Immunology & Pathology, Des Moines University, West Des Moines, IA 50266, USA; 2 School of Medicine and Public Health, University of Wisconsin-Madison, Madison, WI 53705, USA; bsobhani@wisc.edu; 3 Department of Family Medicine and Community Health, University of Wisconsin-Madison, Madison, WI 53705-2700, USA; ejura.salihu@fammed.wisc.edu (E.Y.S.); hannah.wallenkamp@fammed.wisc.edu (H.M.); jordan.edwards@fammed.wisc.edu (J.E.); megan.ringo@fammed.wisc.edu (M.R.); randy.brown@fammed.wisc.edu (R.B.)

**Keywords:** opioids, prolonged opioid use, opioid misuse, opioid use disorder, traumatic injury, trauma patients, risk factors, pain management, prescription opioids, addiction

## Abstract

Traumatic injuries represent a significant public health challenge, affecting millions worldwide annually and necessitating acute pain management that frequently involves the use of opioid analgesics to mitigate discomfort and facilitate recovery. Although opioids remain an integral part of post-traumatic injury pain management, their use exposes trauma survivors to the risk of developing persistent use, misuse, or opioid use disorder (OUD). Pre-injury health determinants, such as age, gender, psychiatric conditions, medical conditions, and substance use history, may interact with injury-related factors to acutely escalate the risk for misuse and addiction. Despite the growing recognition of these potential vulnerabilities, there remains a lack of evidence-based clinical decision support on modifiable and non-modifiable risk factors specific to post-traumatic injury opioid risk trajectories. This review summarizes the literature related to the multifactorial contributors to opioid misuse and addiction following traumatic injury such as patient-level (e.g., demographics, behavioral health), injury-related (e.g., severity, type), and system-level (e.g., prescribing patterns) characteristics. A comprehensive literature search, inclusive of the literature from 1995 to November 2025, was performed in PubMed/MEDLINE, Scopus, and Google Scholar using combinations of terms related to “opioids,” “misuse,” “addiction,” “trauma,” and “injury.” Search keywords and operators were developed in collaboration with a university librarian. Reference lists of articles were searched and synthesized. Case reports, case series, editorials, mini-reviews, letters to editor without original data, and qualitative studies were excluded. The findings of the review are expected to provide insight into clinical-decision making as it relates to the management of pain, pain-related distress and functional impact, and co-occurring conditions that may impact injury-related outcomes and the potential likelihood of substance misuse and addiction.

## 1. Introduction

Traumatic injury is a major public health challenge in the United States and globally. In the U.S. alone, injuries cause more than 43.5 million annual emergency department visits [1]. These account for over 30% of all emergency department visits, demonstrating the burden these injuries have on national health systems. Traumatic injuries commonly call for pain management regimens to optimize comfort and facilitate recovery. Historically, a common choice of pain management in these injuries and surgical procedures associated with traumatic injuries has been opioid analgesics [2]. Different opioids have differing levels of liability for misuse based on lipophilicity, mu-receptor binding affinity, and ease of alternative routes of administration. Hydromorphone, fentanyl, morphine, and oxycodone have abuse potential similar to or exceeding that of heroin [3]. People surviving complex traumatic injury and patients undergoing minor surgery alike are at an increased risk of persistent opioid use, opioid misuse, and opioid use disorder (OUD) due to a variety of potentially addressable predictors [4].

When assessing the current state of opioid use in the U.S., historical changes in opioid prescribing and access to opioids must be concurrently addressed. The current opioid crisis can be traced back to the 1990s during which pharmaceutical companies pushed physicians to consider pain the fifth vital sign and liberally treat issues with their “non-addictive” opioid formulations [5]. Around 2010, heroin prices dropped around the same time prescription related opioid overdose deaths were declared to be an epidemic. In response to these trends and related expert guidance aiming to tame the crisis, physicians became increasingly cautious in prescribing these medications, which contributed to a shift where heroin became the leading cause of opioid-related overdose deaths [6]. This trend continued until 2016 with the rise of fentanyl, a synthetic opioid up to 50× stronger than heroin [7]. Opioid-related overdose deaths have been rising since then, with around 79,358 deaths in 2023 [8].

It is important to define these different types of adverse opioid outcomes in the context of prescriptions for traumatic injury pain control. Persistent opioid use has some variation in its definition by study but is generally defined by the initial dispensation of opioids for pain management following a traumatic injury and at least one refill three months or longer after initial dispensation [9]. Opioid misuse in the context of following traumatic injury is defined as the use of prescription opioids in a different way than prescribed [10]. Finally, opioid use disorder (OUD) is defined as the chronic use of opioids that leads to clinically significant distress or impairment and often is considered addiction [1]. A clear presentation of these described and utilized terms appears in Table 1.

Persistent opioid use is associated with increased morbidity, transition to opioid misuse, the development of mental health disorders, and high-cost healthcare utilization (i.e., ER, hospital) [11,12]. As such, the early identification of patients at higher risk of developing persistent opioid use is critical, so that clinicians might engage appropriate preventative measures. There is a current gap in the literature of a consolidated review of risk factors contributing to the aforementioned opioid-related outcomes. Many studies assess risk factors in isolated scenarios (i.e., rib fractures, burns, trauma surgery) but fail to categorize trauma patients as a whole. The limited prospective data and inconsistent definitions of “persistent use” and “misuse” cause additional difficulty in the research and development of this topic.

This review’s objective is to summarize and consolidate predictors of various adverse opioid-related outcomes following traumatic injury. These outcomes include persistent opioid use, opioid misuse, opioid use disorder, opioid-related overdose death, and the transition from prescription opioid use to illicit use and/or injection use. Predictors include, but are not limited to, medical and psychiatric comorbidities, injury-related and treatment-related factors, as well as patient demographics. With the identification of risk factors, clinicians may be more informed in their decision making when prescribing analgesics. This information may also contribute to the future development of screening tools and the implementation of preventative measures may enhance the well-being of trauma patients at large.

## 2. Materials and Methods

This narrative review synthesizes the existing literature on opioid misuse and addiction following traumatic injury. A narrative approach was selected because of the dearth of the empirical literature [13]. Relevant articles were identified through targeted searches of PubMed and Google Scholar using Medical Subject Headings (MeSH) and keyword combinations related to traumatic injury and opioid use, misuse, and addiction. Search terms included opioid-related concepts (e.g., analgesics, opioid, opioid-related disorders, opioids, heroin), trauma-related concepts (e.g., stress disorders, trauma, traumatic, posttraumatic stress disorder), and injury-related concepts (e.g., wounds, injury, injuries). The were two search strings utilized, with one being “(Analgesics, Opioid OR Opioid-related disorders OR Opioid OR Opioids OR heroin) AND (Stress Disorders, Traumatic OR Trauma centers OR trauma OR traumas OR traumatic OR traumatically OR posttraumatic OR PTSD) AND (wounds and injuries OR wound OR wounds OR injury OR injuries OR injured).” The second was “(Analgesics, Opioid” [mesh] OR “Opioid-related disorders” [mesh] OR Opioid*[tiab] OR heroin[tiab]) AND (“Stress Disorders, Traumatic” [mesh] OR “Trauma centers” [mesh] OR trauma*[tiab] OR posttraumatic*[tiab] OR PTSD[tiab]) AND (“wounds and injuries” [mesh] OR wound*[tiab] OR injur*[tiab]).” The last database search was completed on 11 November 2025. Studies published between 1995 and 2025 were eligible for inclusion if they reported original research involving adult populations aged18 years and older, were published in peer-reviewed journals, and were written in English. All articles from the search were compiled in a shared document for the researchers to review. Five researchers screened the article titles and abstracts for relevance to opioid-related outcomes following physical traumatic injury. The articles were reviewed and tracked in a shared excel spread that assessed which of the mentioned criteria were met. Researchers then reviewed each article and recorded if they met criteria within the excel sheet. Consistent with narrative review methodology, findings were synthesized interpretively and examined thematically to identify patterns, points of convergence and divergence, and gaps in the existing literature [14,15]. Any conflicting information related to inclusion criteria was brought before the entire research team for further review and decision making at routine meetings. Likewise, the entire research team reviewed the synthesis and interpretation of all articles to limit any variability between researchers reviewing said articles. Any conflicting interpretation was given extra review time by the entire team to limit inter-rater reliability concerns.

## 3. Results

### 3.1. Sociodemographic and Psychosocial Risk Factors

Sociodemographics play a significant role in the risk, onset, severity, and outcomes of health and mental health diagnoses, including substance misuse and substance use disorders (SUDs). Green et al. [16] found sociodemographic factors to be the strongest predictors of substance use initiation by early adolescence, citing associations with race, religion, household income, and parental education, amongst others. Similarly, an analysis of the 2011–2019 National Survey on Drug Use and Health revealed associations between age, sex, race, and family income with the presence of SUDs in young adults [17]. In their 10-year follow-up of the National Comorbidity Survey, Swendsen and colleagues [18] suggested that the impact of specific sociodemographic factors may vary depending on specific stage of use, but found that age, education, ethnicity, and occupational status remained risk factors across all stages. Socio-demographics also correlate with SUD outcomes, including treatment quality for patients receiving Buprenorphine and the risk of overdose [19,20].

In this review, sociodemographic and psychosocial factors also appeared to play an important role in opioid use outcomes following traumatic injury. Age, sex, race/ethnicity, education, income, occupation, residential region, marital status, social support, and work fear avoidance all carried associations with opioid-related risk. 

Findings surrounding age were somewhat mixed across identified articles, with the majority pointing to a positive relationship with opioid prescription-related risk. Both Baker et al. [21] and Mauck et al. [22] found age to be associated with new persistent opioid use (NPOU) in hospitalized trauma patients, defined as patients who had no opioid prescription in the year prior to injury, had 1+ opioid prescription fills within two weeks post-discharge, and had 1+ opioid prescription fills 90–180 days post-discharge. The regression model employed by Baker et al. [21] found younger patients aged 18–29 to have lower odds of NPOU compared to patients aged 30–39, 40–49, 50–59, and 60–64. Mauck et al. [22] utilized a comparable model to examine associations with age, both in their overall cohort and in injury-stratified sub-cohorts, finding advancing age to be associated with increased odds of NPOU in their overall cohort. Age associations differed between sub-cohorts, with the burn injury with no graft, open reduction and the internal fixation of long bone fracture, and motor vehicle collision sub-cohorts all generally carrying significant advancing age associations, while the burn injury with graft sub-cohort generally had no significant associations. Additionally, several articles identified age as a risk factor for chronic opioid usage. When examining the demographic characteristics of patients with long-term opioid use (≥90 days of continuous usage) versus no use, Lyons and colleagues [23] found the long-term use group to have a higher mean age than the no use group. Von Oelreich et al. [24] analyzed traumatically injured patients and controls from Swedish health registries and found age to be associated with chronic opioid use. Holman et al. [25] identified advancing age as a risk factor for prolonged opioid use in orthopedic trauma patients. Chaudhary et al. [26] found younger patients in the Military Health System Data Repository were less likely to have sustained use one year after injury. Lastly, Guilcher et al. [27] identified ages 40–60 years to have a perceived increased risk of chronic use and ages 40–50 years as having an increased risk of chronic high-dose use in patients with traumatic spinal cord injuries.

Two articles reported an unclear association with age, and one identified an inverse relationship. Qin et al. [28] found that age < 65 years was associated with persistent opioid use in distal radius fracture patients. The large age range encompassed by this finding makes it difficult to differentiate specific relationships; however, this finding appears to potentially contradict the advancing age associations cited in other studies. In their sample of opioid-naïve patients aged ≥60 years who underwent hip fracture surgery, Okike et al. [29] also found younger age was associated with heightened risk for prolonged opioid usage. However, this finding is limited to their sample of patients aged ≥ 60 years and cannot be generalized to younger age brackets. One final study, that by Bongiovanni et al. [30], did find a clear inverse relationship between age and opioid-related risk. The authors conducted a retrospective chart review of trauma patients and matched patients with unintentional overdose deaths from the California death registry, finding that younger age was associated with subsequent overdose death in their multivariate analysis. In summary, aggregating these age-related findings suggests that while older patients may generally be more at risk for developing NPOU and chronic opioid prescription use, younger patients may carry a higher risk of overdose from said prescriptions.

Sex was tied with age as the most frequently discussed sociodemographic risk factor, with ten articles finding an association. The majority of articles linked female sex with heightened opioid-related risk, while two found an association with male sex. Of the three articles focused specifically on NPOU, all found an association with female sex; Baker et al. [21] and Mauck et al. [22] both found female sex to be associated with NPOU in general trauma patients, and Gossett et al. [31] found the same result in ankle fracture patients. Other studies examined risk factors for chronic use, with four finding an association with female sex and one with male sex. Lyons et al. [23] retrospectively categorized trauma patients into groups of chronic use (≥90 days continuous prescription opioid use) or no opioid prescription use after discharge, finding a higher proportion of females in the chronic use group versus the no use group. Benns et al. [32] performed a similar analysis to Lyons et al. [23] but instead defined chronic use as continued opioid prescription use at one-year post-discharge. Female sex was identified as a risk factor during univariate analysis, but this result did not hold significance upon multivariate analysis. Okike et al. [29] and Daoust et al. [33] both investigated chronic use in elderly trauma patients, finding an association with female sex. In their cohort of traumatic spinal cord injury patients, Guilcher et al. [27] were the only ones to find male sex to be associated with chronic use risk. Male sex was also identified by Bongiovanni et al. [30] as a risk factor for overdose, displaying a similar trend to age, where overdose risk exhibits a potentially inverse pattern of NPOU risk and chronic use risk. 

Findings related to opioid-related risk and race and ethnicity are mixed. Baker et al. [21] found that Asian patients had lower odds of NPOU compared to White patients, but found no difference in odds between Black, White, and Hispanic patients. Similarly, Okike and colleagues [29] found that chronic opioid usage was less common among elderly Asian patients in their hip fracture surgery sample aged ≥ 60 years. When examining data from the Military Health System Data Repository, Chaudhary et al. [26] found Black patients to be less likely to have chronic usage one-year post-discharge compared to White patients, but found no difference between White patients, Asian patients, and other races and ethnicities. Beyer and colleagues [34] performed a retrospective cohort study of battle-injured military personnel, examining the onset of both chronic opioid use and opioid abuse. Although no association was found between race/ethnicity and chronic use, White patients had an increased risk of opioid abuse. Along similar lines, Bongiovanni and colleagues’ [30] analysis of drug overdose following traumatic injury found that White patients were at increased overdose risk. 

Socioeconomic factors, including education, income, and occupation, displayed held consistent risk associations across identified articles. Lower education and income levels were associated with NPOU, chronic opioid use, and opioid-related death [21,24,26,35]. Additionally, when analyzing subsequent opioid poisoning in patients who experienced a work-related injury, Carnide et al. [36] found individuals working in certain industries, including construction, materials handling, processing, and machining occupations, to have an increased risk of opioid-related harm. Teaching, managerial, and administrative professions were found to have a decreased risk of harm. 

A few final sociodemographic and psychosocial factors also carried associations, although with more limited evidence. In one study, patients living in the North-East region of the United States were found to have lower odds of NPOU than patients living in other regions of the country [21]. In another study, marriage was associated with chronic opioid use but was not associated with subsequent opioid abuse [34]. Two psychosocial factors, work fear avoidance and social support, were also discussed. In their prospective observational cohort study of workers’ compensation claims, Elmore and colleagues [37] examined various psychological and psychosocial factors and then compared them to long-term opioid use 6 months post-injury. They found that work fear avoidance, or the belief that returning to work will make an injury or associated pain worse, was a significant risk factor for long-term use. Lastly, Baltes et al. [38] explored the role of social support in prescription opioid misuse risk, gauged by the Current Opioid Misuse Measure (COMM). They found that social support significantly predicted misuse risk 6 months post-discharge, and after controlling for pre-injury anxiety and depression, they found that patients with positive misuse scores had lower baseline social support scores. An illustration of all the factors mentioned can be found in Table 2.

### 3.2. Medical Comorbidities

The burden of chronic medical conditions greatly weighs on the American medical system. The CDC reports that 76.4% of US adults have at least one chronic comorbidity, including but not limited to diabetes (14.7% of adults), hypertension (48.1% of adults), tobacco use (11.6% of adults), liver disease (1.8% of adults), and chronic kidney disease (14% of adults) [39,40,41,42,43]. Few articles have examined how preexisting medical comorbidities contribute to opioid use following traumatic injury. These aspects are important to identify and summarize in order to further stratify patient’s risk factors for developing opioid use outcomes after traumatic injury (Table 3).

Benns et al. [32] defined persistent long-term opioid use as continuously refilling prescriptions within the final three months of the first-year post-discharge. In their study, they found that participants with long-term opioid use were more likely to have a history of diabetes and hypertension. Gong et al. [44] defined persistent opioid use as use between days 91 and 365 post-discharge and found that prior diagnoses of liver disease or chronic kidney disease were associated with an increased likelihood of this persistent opioid use. Dalton et al. [45] found, similarly, that those with liver disease were less likely to discontinue their opioids. These three studies did not delve deeper into the various possible explanations for these findings, leaving gaps for future research.

A history of traumatic brain injury (TBI) has been shown to increase the risk of developing various levels of opioid use. Abid et al. [46] reports that somatic symptoms following TBI, such as headache, dizziness, and fatigue, correlate with the development of opioid use disorder. Similarly, Ilie et al. [47] found that those who screened higher for psychological distress following TBI also had higher rates of opioid use. They also acknowledge that psychiatric symptoms and substance use may be a product of risk-taking behaviors and actually be the igniting source for TBI. Lastly, Fonda et al. [48] reported a three-fold increased risk of opioid overdose in veterans with TBI, though this correlation was decreased when adjusting for comorbid psychiatric conditions.

Instead of describing individual comorbidities, some articles combined them into composite scores using various tools. In this review, many articles referred to a Charleson Comorbidity Index (CCI) score to define their participant population’s comorbidity burden. The CCI takes into account a patient’s age and history of myocardial infarction, congestive heart failure, peripheral vascular disease, stroke/transient ischemic attack, dementia, chronic pulmonary disease, connective tissue disease, peptic ulcer disease, liver disease, diabetes, hemiplegia, moderate to severe chronic kidney disease, solid tumors, leukemia, lymphoma, and AIDs. A higher CCI score indicates a higher disease burden. Two articles reviewed, Gossett et al. [31] and Lyons et al. [23], had results indicating that a higher CCI score resulted in longer opioid use. However, one article, Johnston et al. [49], actually indicated that patients with a higher CCI score were less likely to be discharged with opioids and therefore have a shorter course of prescribed opioids. The explanation offered for this pattern was the idea that providers were more aware of the extensive therapy patients with higher CCI scores received and therefore worked to decrease polypharmacy load. Guilcher et al. [27] used John Hopkins’s Adjusted Clinical Groups (ACG) system to calculate aggregated diagnosis groups (ADG) to define their participants’ morbidity burden. The higher the ADG score, the higher the morbidity burden present. They found that chronic opioid use after traumatic spinal cord injury was positively correlated with a higher median ADG score up to a score of 14, at which the risk plateaued. This finding regarding traumatic spinal cord injury was derived from within-group comparison, not comparison across other injury types.

### 3.3. Injury Characteristics and Length of Stay

Severity and type of injury may have a hand in predicting opioid use behaviors following traumatic injury. Naturally, increased severity often sees increased pain, resulting in a greater need for stronger, long-term pain management with opioid analgesics. Bongionvanni et al. [30] investigated characteristics at the time of injury that resulted in a higher rate of opioid overdose. They found these characteristics to be a lower Glasgow Coma Score (GCS), positive drug screening, and a blood alcohol level > 0 at the time of injury. These findings may be confounded by potential pre-injury opioid or alcohol use, predisposing the traumatic injury patient to a higher risk of opioid use disorder. Benns et al. [32], Anstrom et al. [50] and Held et al. [51] reported that a higher injury severity score (ISS), calculated based on location and type of injury, was found to have a higher risk of long-term opioid use. Another study similarly found that an ISS > 9 was associated with long-term risk of opioid abuse [34]. Tilhou et al. [52] investigated spine injury specifically and found a longer length of stay, increased injury severity rating, and intubation to be associated with higher opioid misuse within this specific sub-sample of injury survivors. Lyons et al. [23] reported pre-existing chronic pain as a risk factor for long-term use among those with orthopedic injuries. Guilcher et al. [27] and Heins et al. [53] found that being discharged to a rehab facility was associated with a higher risk of chronic opioid use, and they used this outcome as a proxy for severity of injury. This demonstrates that the type of traumatic injury bears is less important to subsequent opioid use than other pre-existing risk factors and injury-related complications. Length of stay (LOS) may also play a role in opioid use of patients with traumatic injuries. In multiple studies, length of stay has been investigated as a risk factor for its impact on opioid use. A length of stay greater than 6 days has been found to put traumatic injury patients at an increased risk for developing opioid misuse and addiction [52,54]. Mauck et al. [22] found that LOS as little as greater than 24 h was an increased risk for trauma patients developing opioid misuse. Longer hospital stay was even associated with eventual death from opioid overdose in one study [30]. Similar to other risk factors, a longer LOS’s influence is not limited to just misuse and addiction but also can increase risk for persistent opioid use [26,32,50]. Given these findings, traumatic injury patients prescribed opioid analgesic appear to be at risk for adverse opioid outcomes with any LOS greater than 24 h and at even higher risk if LOS extends to a week. However, it is worth considering that LOS is a complex metric with multifactoral influence contributing to it. While LOS may be associated with more severe and complex injuries in trauma patients with increased analgesia needs, it is an important factor to consider. There are numerous injury characteristics and LOS risk factors that impact opioid-related outcomes in trauma patients (Table 4).

### 3.4. Mental Health Diagnoses and Substance Use

Mental health and substance use disorders are frequently encountered throughout the world and are becoming more common. In the U.S. alone, there was more than a 23% increase in patients who received a mental health diagnosis from 2019 to 2023 [55]. In addition, 1 in 5 adults in the US experienced a mental health illness period in 2024 [56]. When looking at substance use disorders, almost 60% of people over the age of 12 reported use of tobacco products, vaping nicotine, using alcohol, or using an illicit drug in the past month [57]. Exposure to substances and regular use naturally creates a higher likelihood of transition to a substance use disorder [58]. This demonstrates not only the prevalence of mental health conditions and substance use in communities, but also the risk for these individuals to develop a substance use disorder. Those with certain mental health diagnoses may have a further increased risk for this. It has been demonstrated in longitudinal studies that those with mood, anxiety, and disruptive behavior disorders have a higher risk of developing subsequent substance use disorders [59]. Likewise, we explored the relationship between mental health and substance use, experiencing a traumatic injury, and how these factors impact opioid use following traumatic injury. Specifically, we looked at whether or not those with prior mental health diagnoses or substance use developed prolonged opioid use, misuse, or OUD following traumatic injury. Table 5 provides a summary of factors examined.

Depression is a common mental health diagnosis and known risk factor for the development of substance use disorder over time on its own [60]. When factoring in those diagnosed with depression who experience a traumatic injury and received opioids for pain management, this risk is evident but multifaceted. Baltes et al. [38] describes that lower social support composite scores and higher depression composite scores are effective predictors for opioid misuse following traumatic injury. While these two factors can be synergistic, this research demonstrates that depression is a significant risk factor for misuse. They also found that depression and social support scores are risk factors for initiation of opioids as patient’s pain management choice over non-opioid analgesics in traumatic injury. Persistent or long-term opioid use following traumatic injury varies by definition but typically involves tracking the number of opioid prescriptions dispensed or months used. Frequently, multiple dispensations of opioids or use longer than 3 months post-injury are used as the standard for long-term or persistent use. Pre-injury depression has been found to be a significant risk factor in the development of persistent opioid use following traumatic injury in multiple studies [23,30,31,45,53,61,62]. Interestingly, there have been a few studies that have examined whether the development of depressive symptoms in association with traumatic injuries leads to persistent opioid use. However, these studies reveal mixed results. Sharififar et al. [63] found that after administering the Beck Depression Inventory (BDI-II), patients using opioids for pain management beyond six weeks were significantly associated with higher scores on the BDI-II. In contrast, Elmore et al. [37] found no significance regarding the association between depressive symptoms and persistent opioid use when examining psychosocial factors. While there is not a large volume of research specifically regarding the impact of developing depressive symptoms alone, this provides an opportunity for future research in patients with traumatic injuries who were prescribed opioids. Another variation in opioid use that has been examined in the context of traumatic injury is opioid use disorder (OUD). This is defined as the chronic use of opioid that leads to significant distress or impairment [12]. Pre-injury depression has also been found to be a significant risk factor for the development of OUD following traumatic injury [64]. There is also evidence that patients with pre-injury depression and anxiety disorders who experience TBIs may be especially at risk for the development of OUD [46]. Pre-injury depression is an established and significant risk factor for developing persistent opioid use, opioid misuse, and OUD following traumatic injuries. This should be considered when navigating analgesics for patients with traumatic injuries.

Anxiety disorders are also extremely prevalent across the globe. Last year, approximately 19.1% of US adults had a formal diagnosis of an anxiety disorder [65]. However, when looking at whether or not evidence exists to suggest it can lead to the development of substance use disorders, this varies compared to other mental health diagnoses like depression. Groenman et al. [60] found that while depression longitudinally can lead to increased risk of developing a substance use disorder, the same risk does not exist for anxiety. Additionally, there is evidence to suggest that this lack of risk extends to patients with an anxiety disorder who experience a traumatic injury and are prescribed opioids [64]. This suggests that traumatic injury patients with anxiety disorders may not have an increased risk of developing opioid misuse or OUD compared to patients with other mental health diagnoses. There remains conflicting evidence here as well. Baltes et al. [38] also found that a higher anxiety composite score is an effective predictor for opioid initiation and misuse following traumatic injury. Overall, there is discord regarding whether pre-injury anxiety is a risk factor for opioid misuse and OUD in traumatic injury patients. This provides an opportunity for future research to investigate these two adverse opioid-related outcomes in the context of anxiety. In contrast, there are multiple studies that demonstrate traumatic injury patients with pre-injury anxiety disorders have an increased risk of developing persistent or long-term opioid use, similar to depression [23,29,31,32,33,61]. This illustrates that while more research may be needed to parse out whether pre-injury anxiety disorders lead to an increased risk of developing opioid misuse or OUD following traumatic injuries, it is a clear risk in terms of persistent use. Similar to depression symptoms, post-injury anxiety symptoms were not found to be an increased risk for persistent opioid use [37]. Pre-injury anxiety is a significant risk factor to be considered clinically for the development of persistent opioid use following traumatic injury.

A less prevalent but well-examined mental health diagnosis in relation to opioid use and traumatic injury is post-traumatic stress disorder (PTSD). PTSD is defined as a mental health diagnosis that may develop following exposure to a traumatic event, characterized by symptoms such as intrusive memories, avoidance behaviors, and negative alterations in cognition and mood lasting more than one month and causing significant distress [66]. PTSD is relevant in the context of traumatic injuries as to its influence on opioid use if it occurs prior to the injury or as a result of it. Several studies evaluated traumatic injury patients for pre-injury PTSD symptoms via questionnaires and found that patients experiencing higher PTSD symptoms were more likely to have long-term opioid use [53]. Another study by Brown et al. [54] found that higher pre-injury PTSD symptoms were associated with an increased risk of developing opioid misuse. Traumatic injuries themselves may be the inciting factor for the development of PTSD, depending on the situation and severity. In a study by Schultebrauks et al. [67], the authors developed a tool that accurately predicted approximately 17% of trauma patients develop PTSD from the injury, which is a significant amount. Given this risk of development, there is also the risk PTSD from the injury may influence opioid use following it. Helmherhorst et al. [62] investigated whether post-traumatic injury PTSD scores via surveys predicted persistent opioid use. They found that a higher PTSD score following the traumatic injury was associated with long-term opioid use. PTSD, whether originating pre-traumatic injury or symptoms appear following the injury, appears to be a risk factor for adverse opioid outcomes and should be monitored.

Substance use and substance use disorders are important factors to examine in the context of how they influence opioid use following traumatic injury. While the number of substances use disorder diagnoses has remained relatively stable over the past few years, there are over 48 million people in the US currently diagnosed with one [57]. This inevitably leads to a proportion of traumatic injury patients who have pre-injury substance use or substance use disorder. There have been studies examining the impact of substance use on opioid use following traumatic injury. Many of these examine substance use in general as a risk factor, defined as any history of substance use pre-injury that does not meet criteria for substance use disorder (SUD), as well as SUD itself. It has been demonstrated that tobacco use, pre-injury opioid use, pre-injury non-opioid substance use and dependence, substance use disorder, and particularly alcohol use disorder all have increased risk for leading to persistent or long-term opioid use following traumatic injury [23,28,29,30,31,32,34,44,45]. Lapidus et al. [68] also found that whether developing persistent opioid use or not, the history of pre-injury substance use was associated with more opioid refills and a higher likelihood of potentially inappropriate prescribing (PIP) in trauma patients. Overall, a history of substance use or substance use disorder appears to put trauma patients at a higher risk for opioid analgesic refills and developing long-term use. Any use of opioid analgesics carries with it the risk of overdose (OD). This creates the possibility of OD in trauma patients who were prescribed opioids. There have been some specific risk factors within substance use that may put trauma patients at risk for an OD. There is evidence that a history of smoking tobacco creates a higher risk for OD in traumatic injury patients [69]. As mentioned previously, Bongiovanni et al. [30] examined risk factors for drug poisoning following traumatic injury and found that a blood alcohol content greater than 0 at the time of injury led to an increased risk of opioid OD in trauma patients who had been prescribed them. This implies that alcohol use history is an important risk factor to consider when prescribing. Both this and tobacco use are expected but important considerations as to increased risk for OD and prolonged opioid use when prescribing opioid analgesics for traumatic injury.

### 3.5. Pain-Related Coping, Function, and Self-Efficacy

Pain-related coping, function, and self-efficacy are important considerations that can influence how patients recover from traumatic injury and utilize opioid analgesics. There is a limited amount of research that exists around these topics, making available evidence valuable. Table 6 consolidates findings around these factors. Pain-related coping refers to how patients view, interpret, and manage their pain. It can be assessed through questionnaires such as the “Pain Catastrophizing Scale” (PCS). Brown et al. [54] utilized this scale to investigate how pain coping influences opioid use following traumatic injury. They found that among other risk factors, patients who had poor coping skills or higher scores on the PCS were more likely to develop opioid misuse and OUD six months after hospital discharge. This supported additional studies which showed impaired coping skills, symptoms of PTSD, and pain anxiety were observed in patients with persistent opioid use [62]. Pain-related coping skills are a reliable predictor of adverse opioid outcomes when assessed in traumatic injury patients.

Pain-related function or function while in pain is another measure to consider for patients with traumatic injuries. It examines the impact of physical function of those who experienced a traumatic injury and can influence opioid analgesic use. Traumatic injuries and their recovery process can place physical limitations upon patients and cause pain, which can lead to mental stress originating from their injury. Kessler et al. [70] found that when following opioid use in trauma patients, injury-related stress was the largest predictor of misuse. Expanding upon this, Elmore at al. [37] found that work fear avoidance and anxiety due to the injury created stress in patients and were associated with long-term opioid use as mentioned previously. The physical burden and compounding of multiple injuries or surgeries in trauma patients is also a factor to consider. As previously stated, Abid et al. [46] found that patients with continuous somatic symptoms of TBI and those with multiple surgeries had a higher risk of long-term opioid use. This makes pain-related function a key piece in the evaluation of post-traumatic injury patients.

Self-efficacy is another patient function that has been studied in the context of traumatic injury and opioid use. It evaluates what actions the patient takes and what supports they utilize in recovery. It is a measure of interest as it can be used as a predictor for the degree of opioid use following traumatic injury. When examining strength and the utilization of social support, a lack thereof has been found to cause an increased risk of traumatic injury patients initiating prescription misuse of opioids [38]. Additionally, as mentioned before, a lack of social support has not only been found lead to an increased risk of misuse, but also an increased risk of addiction at 6 months [54]. Self-efficacy can also be influenced inversely by opioid use following traumatic injury. Helmerhorst et al. [62] found that patients who continued using opioids after surgery tended to experience more psychological stress, leading to less effective coping strategies. Following a traumatic injury, discharge location may influence opioid use as well due to support factors. Qin et al. [28] discussed that discharge to a location other than home was associated with persistent post-operative opioid use. This may be due to differences in pain management procedures at certain nursing or rehabilitation facilities. It may also result from a lack of patient’s self-efficacy in pain management and care planning, evidenced by not being able to be discharged home. Self-efficacy is a multifactorial influence to be considered in opioid prescribing following traumatic injury.

### 3.6. Trends in Opioid Prescribing

Based on this review search, we see qualitative evidence that current opioid prescribing guidelines lead to varying pain management outcomes in trauma setting. One study in particular highlighted a disconnect between broad regulatory recommendations and the nuances of the clinical needs of individuals with complex, injury-related pain [71]. Additionally, provider and staff perspectives from multiple sites emphasize the necessity for trauma-specific opioid prescribing frameworks to better align practice with evolving trends in acute and long-term pain management following traumatic injury [71]. Another study brought to light a current gap in opioid prescribing, namely risk mitigation strategies such as naloxone co-prescription, and the relationship this has to overdose risk in trauma populations [72]. The study demonstrates that a high proportion of acutely injured trauma patients possess one or more risk factors for unintentional opioid overdose, emphasizing that these populations usually exhibit significant risk for substance misuse along with being prescribed opioids [72]. With this being said, another article found that although opioids remained a central component of acute pain management after trauma, fewer than half of opioid-naive trauma patients were discharged with opioid prescriptions, showing a potential shift toward reduced discharge opioid prescribing practices altogether in this population [49]. The study also found that certain clinical factors, such as age, were associated with a lower likelihood of an opioid being part of the patient’s discharge plan [49]. One study, in particular, looked at a subset of the traumatic injury population: rib fracture patients. This study in particular revealed that only a minority of patients with rib fractures were prescribed opioids, which seems to align with other sources showing that the rate of initial opioid prescribing in this trauma population is decreasing, and seems to be mostly dependent on prior opioid exposure rather than injury severity [45]. Another study showed similar findings post-discharge, particularly that factors such as prior substance use and mental health disorders were associated with an increased likelihood of opioid refills after traumatic injury [68]. Overall, the reviewed literature suggests that opioid prescribing practices in trauma care are increasingly evolving.

### 3.7. Efficacy of Screening Tools

Screening tools exist and are starting to be developed and utilized for the prevention of adverse opioid-related outcomes. However, these have primarily been studied in the setting of chronic pain. As such, this is an area requiring further development and validation, particular regarding traumatic injury, as well as pragmatic and implementation research; it is beyond the scope of this review. The most common assessments mentioned were the Screener for Opioid Assessment for Patients with Pain-Revised (SOAPP-R) and the Opioid Risk tool (ORT). Many times, informal assessments or calculations were used to ask about early requests to refill prescriptions, perceived pain, and other subjective, non-validated assessments. Morphine Milligram Equivalent (MME) scores were also identified as common patient assessment measures and were only completed while in inpatient services and not upon discharge [51,72,73]. While much work has been done on chronic non-cancerous pain, much of that analysis and assessment is outside of the scope of this review [6].

The SOAPP-R was used in studies in the emergency department setting and it was speculated that it could be a useful tool for screening [74,75]. About one-third of patients were considered high risk in this population. The assessment was judged to be easy to administer and timely in completion for patients, taking less than 5 min to complete, and was completed with the help of study staff. For trauma populations, this assessment could be considered given the low requirements of effort from staff and patients.

The ORT was also used across a number of studies, both completed studies and early protocol papers [76]. In some studies, they found that the Brief Risk Interview (BRI) was better at detecting aberrant medication-related behavior and overall had better sensitivity and predictive accuracy compared to the ORT and SOAPP-R [77]. The latter are written questionnaires filled out by patients. This assessment was validated in trauma populations (though specifically in the emergency department setting), and so could represent a potential tool in these populations [78]. Looking at current risk factors for potential opioid misuse by people experiencing trauma, major risks were identified in populations who also experienced higher stress [70].

In general, these papers provided insight into potential tools for screening related to opioid-associated risk, though there was no one assessment deemed ‘best’ at assessing for opioid misuse potential and there were none specifically studied with long-term follow up in survivors of traumatic injury. However, the ORT was cited as the most regularly used assessment alongside other risk factors, such as stress, the previous use or misuse of substances, and the strength of prescription. All mentioned screenings are tools for potential consideration in trauma populations, though research and validation particular to follow up after hospitalization for injury are lacking. However, they represent potentially pragmatic screens, given their efficiency and low participant and staff burden.

## 4. Discussion

This narrative review synthesizes the existing literature to highlight and consolidate risk factors for persistent opioid use, misuse, and OUD following traumatic injury. Findings illustrate a multifaceted interplay of medical, psychological, injury-related, and treatment-related predictors for opioid-related outcomes. The findings underscore that while opioid analgesics remain a cornerstone for acute pain management in trauma patients, several factors elevate the risk for adverse opioid outcomes. Sociodemographic factors, encompassing age, sex, race/ethnicity, and other psychosocial elements like education, income, and social support, reveal nuanced associations with adverse opioid outcomes. Advancing age is frequently linked to elevated risks of new persistent opioid use and chronic use across various trauma cohorts, potentially reflecting age-related pain complexities or prescribing biases, though younger age heightens overdose mortality [21,27,29,30]. Female sex predicts persistent and chronic use in general and specific injury populations in direct analyses but not multivariate assessments. Male sex is associated with chronic use in spinal cord injuries and overdose in broader trauma samples [21,22,23,27,29,30,32,33]. Findings on race/ethnicity demonstrate mixed results. Asian patients often show lower odds of persistent opioid use compared to White patients, White patients show a higher risk of abuse and OD, and Black patients demonstrate reduced chronic use in military contexts [21,26,29,30,34]. This highlights potential disparities in access or cultural factors. These sociodemographic insights emphasize the role of broader social determinants in shaping adverse opioid outcomes and warranting tailored approaches in clinical risk stratification.

Medical comorbidities such as diabetes, hypertension, liver disease, chronic kidney disease, and TBI consistently emerge as risks for persistent opioid use, potentially due to compounded pain, the complexity of managing polypharmacy, or altered pharmacokinetics that can contribute to prolonged opioid dependence [39]. Additionally, higher comorbidity indices like the Charlson Comorbidity Index (CDI) or Aggregated Diagnosis group (ADG) scores correlate with chronic opioid use [23,27,31,49]. These associations emphasize the need for integrated risk stratification that accounts for preexisting health burdens in pain management of trauma patients.

Injury characteristics and hospital length of stay (LOS) appear to further amplify risk. Severe injuries determined by injury severity scores, orthopedic injuries, lower Glasgow coma scores, and extended hospitalization (greater than 24 h and particularly greater than 6 days) were linked to higher rates opioid misuse and persistent use [22,26,30,32,34,50,51,52,53,54]. This may reflect how prolonged opioid exposure during inpatient care or incompletely addressed pain from complex recoveries, compounded by proxies for severity such as discharge to rehabilitation facilities. Mental health diagnoses, including pre-injury depression, anxiety, and post-traumatic stress disorder (PTSD), demonstrate strong predictive value for persistent opioid use, misuse, and opioid use disorder (OUD) [23,29,31,32,33,38,45,53,54,60,61,62,64,67]. This aligns with a pre-injury history of substance use and substance use disorders [23,28,29,30,31,32,34,44,45,68,69]. However, there is conflicting evidence on pre-injury anxiety. This is a fantastic opportunity for future research to standardize definitions and parse out this risk factor. Particularly, there is a need to investigate the risk of pre-injury anxiety for the development of persistent use, opioid misuse, or OUD, as this is where conflicting findings exist. Given the prevalence of anxiety in communities, it could serve as a valuable target of trauma and opioid research. Depression and PTSD symptoms, either when they emerge pre-injury or are injury-induced, particularly increase vulnerability to adverse opioid outcomes, potentially through maladaptive coping mechanisms or pain perception. Substance use disorders, including those relating to tobacco and alcohol, not only predict the initiation of opioid refills and long-term use but also elevate the risk of overdose, illustrating a relationship where prior misuse predisposes traumatic injury patients to subsequent opioid escalation [23,28,29,30,31,32,34,44,45,68,69].

Pain-related coping, function, and self-efficacy represent psychosocial dimensions that influence opioid outcomes with poor coping skills (high pain catastrophizing scale scores), injury-related stress, and limited social support associated with misuse and OUD [28,37,38,46,54,62,70]. These factors highlight the role of psychological resilience and social support in recovery, where physical limitations from multiple surgeries or somatic symptoms resulting from injuries like TBI may exacerbate dependence. Trends in opioid prescribing reveal an evolving landscape, with reductions in discharge prescriptions for opioid-naive patients and rib fracture cases driven by an increasing awareness of overdose risk and factors like prior substance use [45,49,68,71,72]. However, gaps persist in trauma-specific guidelines, naloxone co-prescribing, and alignment with individual needs, suggesting a disconnect between regulatory frameworks and clinical nuances. Screening tools such as the Opioid Risk Tool (ORT), Screener and Opioid Assessment for Patients with Pain-revised (SOAPP-R), and Brief Risk Interview (BRI) show promise for identifying high-risk patients, though no single tool dominates, and their validation in trauma populations will continue to grow. Opportunity exists for future research to validate these existing tools in trauma populations, as well as to develop and test new tools.

This review offers several strengths that advance the understanding of opioid-related outcomes in trauma patients. First, it consolidates multifactorial predictors across patient-, injury-, and system-level domains to address key gaps in the literature. Additionally, it synthesizes these factors across a variety of studies into a cohesive framework for trauma populations. The broad search strategy using diverse search string terms and multiple search engines provides enhanced comprehensiveness of findings. By identifying modifiable and non-modifiable risk factors, it helps to provide condensed and comprehensive components that may put patients at higher risk of adverse opioid-related outcomes. Therefore, it can support trauma care optimization amid the ongoing opioid crisis.

Several limitations identified during the writing process of this review warrant consideration. It was observed that there are heterogenous definitions of persistent or long-term opioid use. Heterogenous definitions are an important consideration of this review in terms of interpretation, with variation in definitions of opioid-related outcomes. In particular, variation was present across the definition of persistent opioid use or long-term opioid use. While studies had different time points or refill numbers they deemed as persistent use or long-term use, they were similar across these studies. Nevertheless, this should be considered as a limitation upon the interpretation and grouping of similar results across studies as no uniform definition of persistent opioid use currently exists. This complicated direct comparisons and meta-analytic potential, underscoring the need for standardized metrics in future research. There was also a predominance of articles meeting criteria that were both retrospective and U.S.-based. This restricts causal inference and the temporal sequencing of risk factors. It also potentially limits generalizability to international contexts with differing healthcare systems, injury epidemiology, or opioid access. The largest volume of these articles and data is from the U.S. given the nature of the opioid crisis. Most of these studies tend to rely on registry-based data from patients, limiting study design due to some factors being out of control; this can also limit the direct comparison of these results. In addition, not all mental health diagnoses or patterns of opioid use were addressed, as the scope of the review was determined by the volume and nature of evidence currently available in the literature. Consequently, important variations may not be fully represented. Within mental health diagnoses, there is specifically an opportunity for more research on how injury-induced symptoms may impact opioid outcomes due to conflicting evidence and a lack of volume. Another consideration is that not all risk factors have been assessed to equal amounts, leading to some being more established than others. This impacts comparison across studies, populations, and other nations. This limitation is also addressed by highlighting the number of articles discussing each risk factor in the tables. There was also the deliberate exclusion of some qualitative evidence in an attempt to prioritize quantitative risk associations amenable to thematic aggregation. However, this potentially overlooks nuanced patient experiences like certain social barriers. In an effort to balance this, sociodemographic and psychosocial risk factors were thoroughly reviewed. During article selection and review, we attempted to reduce inter-rater reliability by using standardized and tracked criteria to be met. Additionally, synthesis and interpretation were done and reviewed by the entire team in an effort to limit this as well. However, there still exists a possibility of inter-rater reliability in selection and synthesis due to the involvement of multiple reviewers.

For future research, prospective, multicenter trials are essential to test interventions’ efficacy using standard definitions of persistent and long-term opioid use and appropriate gold standards for the development of use disorders. Standardized definitions for these terms are also needed to enable meta-analyses. Studies should prioritize under-investigated areas, such as injury induced mental health impacts and transitions to illicit opioid use. It should also consider incorporating multinational cohorts to enhance generalizability beyond U.S. centric data. Randomized controlled trials evaluating trauma-specific screening algorithms or bundling of preventive measures based upon these identified risk factors would provide causal evidence. In addition to the need for research incorporating these risk factors into screening algorithms or bundles, there are also trials needed on adverse opioid-related outcomes when these risk factors are taken into consideration when prescribing. Research including qualitative components could be helpful to capture patient perspectives on barriers to inform holistic preventative strategies. At minimum, we recommend clinicians perform a comprehensive history review and consider these aforementioned risk factors and trends when choosing pain analgesia for traumatic injury patients. By addressing these gaps, clinicians can better identify at-risk patients early, potentially mitigating the transition from acute pain management to adverse opioid outcomes, and reduce the societal burden of the opioid epidemic.

This review offers consolidation and comprehensive synthesis of risk factors of adverse opioid outcomes in traumatic injury patients. The goal of providing these risk factors is to offer insight to clinicians on considerations they should weigh when reviewing a traumatic injury patient’s history and prescribing analgesia. In addition, it may provide the identification of risk factors that can be further investigated in research alone or inform and be incorporated into preventative screening or risk tools for traumatic injury patients.

## 5. Conclusions

Specific patient risk factors pose a significant risk for persistent opioid use, misuse, and addiction in those with traumatic injuries. These include, but are not limited to, medical comorbidities, injury severity, mental health diagnoses, substance use histories, pain coping mechanisms, and evolving prescribing practices. This review consolidates evidence of risk factors like, but not limited to, pre-injury depression. This can be considered and utilized to inform preventive measures, e.g., enhancing screening and multidisciplinary interventions to protect vulnerable patients. Findings from this review may help improve screening, not only through informing clinicians of important considerations when prescribing opioids but also suggesting risk factors that may be utilized in developing risk screening tools or algorithms. Despite some advancements in opioid stewardship, variation in definitions and the limited prospective data underscore the need for further research for refining trauma specific guidelines and screening tools. The evidence provided in this review hopes to support these efforts and goals in future research. Ultimately, integrating these insights into clinical practice, screening, and guidelines may reduce adverse opioid outcomes, improve patient recovery, and contribute to curbing the opioid crisis.

## Figures and Tables

**Table 1:** Opioid-related risk term definitions.

Risk Term	Definition
Chronic Opioid Use Long-Term Opioid Use	Consistent use of prescription opioids for three months or longer. Typically used in the context of prescription use for chronic pain conditions.
Persistent Opioid Use (POU)	Initial dispensation of opioids for pain management following a traumatic injury and at least one refill three months or longer after initial dispensation. Typically used in the context of prescription use following hospitalization or surgery.
New Persistent Opioid Use (NPOU)	Initial dispensation of opioids for pain management following a traumatic injury and at least one refill three months or longer after initial dispensation in a patient with no history of prescription opioid use.
Opioid Use Disorder (OUD)	Chronic use of opioids that leads to clinically significant distress or impairment and often is considered addiction.
Opioid Misuse	Use of prescription opioids in a different way than prescribed.
Opioid-Related Overdose	Overdose involving prescription or illicit opioids.

List of opioid-related risk terms with corresponding definitions. These terms were utilized across many studies and are reflected in either standard definition or most utilized definition. Note: There is some variation across articles in exact definitions utilized such as persistent opioid use (POU).

**Table 2:** Sociodemographic and psychosocial factors and opioid-related risk.

Risk Factor	No. of Articles Discussed	Result Consistency Across Articles	Risk Associations (No. of Articles)
Age	10	Mixed	Older age—increased risk (7) Younger age—increased risk (2) Age < 65—increased risk (1)
Sex	10	Mixed	Female—increased risk (8) Male—increased risk (2)
Race/Ethnicity	5	Mixed	White—increased risk (2) Asian—decreased risk (2) Black—decreased risk (1)
Education/Income	4	Consistent	Lower education/income—increased risk (4)
Occupation	1	-	Select industries ^a^—increased risk (1) Teaching, managerial, & administrative roles—decreased risk (1)
Region	1	-	North-East of US—decreased risk (1)
Marital Status	1	-	Married—increased risk (1)
Work Fear Avoidance ^b^	1	-	Higher work fear avoidance—increased risk (1)
Social Support	1	-	Lower social support—increased risk (1)

Examined sociodemographic and psychosocial risk factors and associations examined across studies. ^a^ Construction, materials handling, materials processing, machining, transportation, utilities, equipment operating, service, medicine and health, sales, manufacturing, forestry, fishing, trapping, farming, horticultural, and animal husbandry [35]. ^b^ Work fear avoidance: concern that work will make an injury and its associated pain worse [36].

**Table 3:** Medical comorbidities and opioid-related risk.

Risk Factor	No. of Articles Discussed	Result Consistency Across Articles	Risk Associations (No. of Articles)
Traumatic Brain Injury (TBI)	3	Consistent	Occurrence of TBI—increased risk (1) Presence of somatic symptoms following TBI—increased risk (1) Higher psychological distress following TBI—increased risk (1)
Charleson Comorbidity Index (CCI) ^a^	3	Mixed	Higher CCI score—increased risk (2) Higher CCI score—lower likelihood of receiving opioid prescription at discharge (1)
Liver Disease	2	Consistent	Increased risk (2)
Kidney Disease	1	-	Increased risk (1)
Diabetes	1	-	Increased risk (1)
Hypertension	1	-	Increased risk (1)
Adjusted Clinical Groups (ACG) system ^b^	1	-	Higher ADG score—increased risk (1)

Examined medical comorbidity risks and associations across studies. ^a^ Higher CCI scores indicate higher comorbid disease burden. ^b^ Higher ACG scores indicate higher comorbid disease burden.

**Table 4:** Injury characteristics, length of stay, and opioid-related risk.

Risk Factor	No. of Articles Discussed	Result Consistency Across Articles	Risk Associations (No. of Articles)
Length of stay (LOS)	7	Consistent	LOS > 6 days—increased risk (2) LOS > 24 h—increased risk (1) Longer LOS—increased risk (4)
Injury Severity Score (ISS) ^a^	5	Consistent	Higher ISS—increased risk (4) ISS > 9—increased risk (1)
Discharge to rehab facility	2	Consistent	Increased risk (2)
Intubation	1	-	Increased risk (1)
Pre-existing chronic pain	1	-	Increased risk (1)
Glasgow Coma Score (GCS) ^b^	1	-	Lower GCS—increased risk (1)
Drug Screen Results	1	-	Positive drug screen—increased risk (1)
Blood Alcohol Level (BAC) at time of injury	1	-	BAC level > 0 at time of injury—increased risk (1)

Examined injury characteristics and length of stay risk factors and associations examined across studies. ^a^ Higher ISS indicates greater injury severity. ^b^ Higher GCS indicates lower impairment of consciousness.

**Table 5:** Mental health diagnoses, substance use, and opioid-related risk.

Risk Factor	No. of Articles Discussed	Result Consistency Across Articles	Risk Associations (No. of Articles)
Depression	12	Mixed	Depression symptoms post-injury—increased risk (2) Pre-existing depression—increased risk (9) Depression symptoms post-injury—no association (1)
Substance Use	10	Consistent	Pre-injury substance use and substance use disorders—increased risk (9) BAC level > 0 at time of injury—increased risk (1)
Anxiety	9	Mixed	Pre-existing anxiety—no association (2) Anxiety symptoms post-injury—increased risk (1) Pre-existing anxiety—increased risk (6)
Post-Traumatic Stress Disorder (PTSD)	3	Consistent	PTSD symptoms post-injury—increased risk (2) PTSD symptoms pre-injury—increased risk (1)

Examined mental health diagnoses and substance use risk factors and associations examined across multiple studies. Note: Not all mental health or substance use diagnoses may be included due to prevalence of existing data.

**Table 6:** Pain-related coping, function, self-efficacy, and opioid-related risk.

Risk Factor	No. of Articles Discussed	Result Consistency Across Articles	Risk Associations (No. of Articles)
Pain-related functioning	3	Consistent	Injury-related stress—increased risk (1) Work fear avoidance ^a^—increased risk (1) Presence of somatic symptoms following TBI—increased risk (1)
Self-Efficacy	3	Consistent	Lack of social support—increased risk (1) Higher psychological stress—increased risk (1) Discharge to location other than home—increased risk (1)
Pain Coping & Pain Catastrophizing	2	Consistent	Higher pain catastrophizing—increased risk (1) Impaired pain coping skills—increased risk (1)

Pain-related coping, function, and self-efficacy examined as risk factors and associations across studies. ^a^ Work fear avoidance: concern that work will make an injury and its associated pain worse [36].

## Data Availability

No new data were created or analyzed in this study.
